# Rescue of a H3N2 Influenza Virus Containing a Deficient Neuraminidase Protein by a Hemagglutinin with a Low Receptor-Binding Affinity

**DOI:** 10.1371/journal.pone.0033880

**Published:** 2012-05-01

**Authors:** Mathilde Richard, Alexandra Erny, Bertrand Caré, Aurélien Traversier, Mendy Barthélémy, Alan Hay, Yi Pu Lin, Olivier Ferraris, Bruno Lina

**Affiliations:** 1 Laboratoire Virologie et Pathologie Humaine (VirPath), EMR 4610 – UCBL, Faculté de Médecine RTH Laennec, Université de Lyon, Lyon cedex, France; 2 Université de Lyon, CNRS, INRIA, INSA-Lyon, LIRIS, UMR5205, F-69621, Lyon, France; 3 The National Institute for Medical Research, Mill Hill, London, United Kingdom; University of Minnesota, United States

## Abstract

Influenza viruses possess at their surface two glycoproteins, the hemagglutinin and the neuraminidase, of which the antagonistic functions have to be well balanced for the virus to grow efficiently. Ferraris et al. isolated in 2003–2004 viruses lacking both a NA gene and protein (H3NA- viruses) (Ferraris O., 2006, Vaccine, 24(44–46):6656-9). In this study we showed that the hemagglutinins of two of the H3NA- viruses have reduced affinity for SAα2.6Gal receptors, between 49 and 128 times lower than that of the A/Moscow/10/99 (H3N2) virus and no detectable affinity for SAα2.3Gal receptors. We also showed that the low hemagglutinin affinity of the H3NA- viruses compensates for the lack of NA activity and allows the restoration of the growth of an A/Moscow/10/99 virus deficient in neuraminidase. These observations increase our understanding of H3NA- viruses in relation to the balance between the functional activities of the neuraminidase and hemagglutinin.

## Introduction

Each winter, influenza epidemics have a considerable impact on the population in terms of morbidity and mortality. Influenza A viruses present at their surface two glycoproteins, the hemagglutinin (HA) and the neuraminidase (NA). The HA allows the virus to bind to the host cell through its binding to cellular sialylated glycoconjugates and recognizes two principal types of sialic acid receptors : sialic acids bound to galactose by a α2.3 link (SAα2.3Gal) or by a α2.6 link (SAα2.6Gal). The NA, through its sialidase activity, releases progeny virions from host cells and has both α2.3 and α2.6 receptor cleavage capacity.

The two proteins, HA and NA, have apparent antagonistic functions : the HA is a receptor binding protein and the NA a receptor destroying enzyme. However, these roles are complementary and their activities must be well balanced for the virus to exhibit good fitness. The counterbalancing of defects in the NA by decrease in HA receptor binding affinity is a well known mechanism observed in drug resistant NA mutants [1], [2], [3], in mutants lacking the coding capacity for the NA gene [4], [5], [6] or in stalkless NA mutants [7], [8]. Equilibration of the balance between the HA and NA has been shown to be necessary for the virus to adapt to a new host [9], [10] or after genetic reassortment [11], [12] [13].

Ferraris et al. reported the isolation in 2003–2004 of four neuraminidase inhibitor-resistant mutants lacking the entire NA gene and protein (H3NA- viruses) [14]. Further studies indicated the essential role of the HAs of two H3NA- viruses (A/Lyon-CHU/26430/03, Genbank JF421757 and A/Reunion/586/04, Genbank JF421758) in compensating for the absence of NA [15]. In the present study, we observed that the HA of the H3NA- viruses has low affinity for SAα2.6Gal receptors and no detectable affinity for SAα2.3Gal receptors, rendering the virus less dependent on NA activity.

In a previous study, we identified a recombinant A/Moscow/10/99 (H3N2) virus deficient in NA activity which contains two structural mutations in the NA active site (E119D/I222L) [16]. This combination of two mutations has however not been observed in the clinic. The E119D mutation has been observed *in vitro* in N2 subtype viruses following passage under zanamivir selection [1], [17]. The I222L mutation has been observed in H5N1 viruses isolated from birds conferring a reduced sensitivity to oseltamivir [18]. The virus possessing the double mutation in NA was able to grow in MDCK cells only in the presence of complementary bacterial NA activity, suggesting that the activity of the NA was abolished by the double mutation E119D/I222L. In this paper, we confirmed that the E119D/I222L mutation in NA has a detrimental effect on the NA activity and showed that the low sialic acid receptor affinity of the HA of the H3NA- viruses was able to counterbalance the deficiency in NA activity caused by the combination of the two E119D and I222L mutations, but not the absence of a NA gene.

## Results

### Differences in HA Affinity for Sialic Acid Receptors

**Figure 1:**
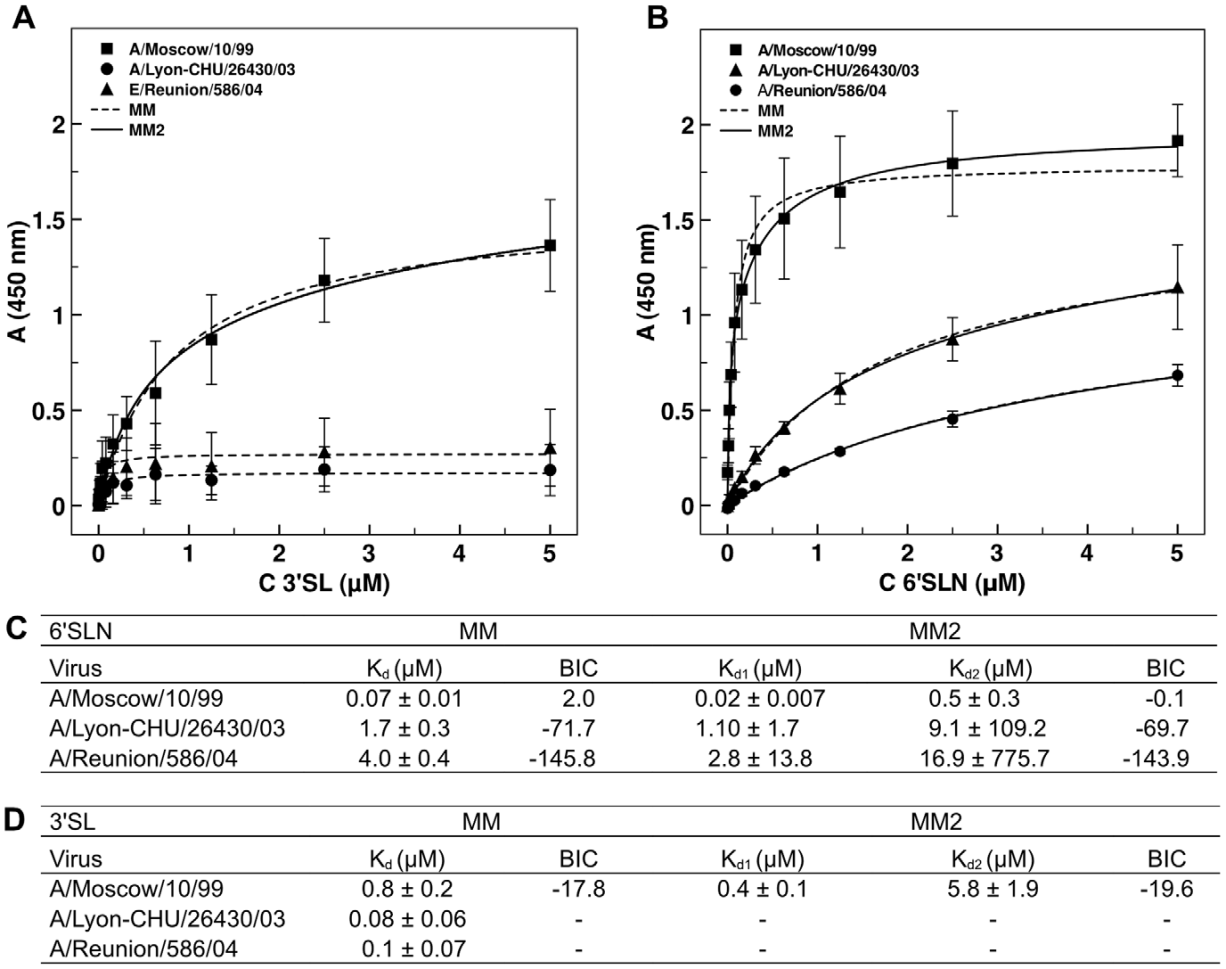
Affinities of the HAs of the H3NA- viruses for sialylglycopolymers. Binding curves were fitted with the two models MM and MM2 for A/Moscow/10/99, A/Lyon-CHU/26430/03 and A/Reunion/586/04 viruses with 6′SLN receptors (A) and 3′SL receptors (B). The values of the dissociation constant (K_d_) of the three viruses for 6′SLN receptors (C) and 3′SL receptors (D) calculated from the two models (MM and MM2) are indicated. The values correspond to the mean +/– standard deviation calculated from three independent assays. The Bayesian Information Criterion (BIC) is indicated for each regression.

We hypothesized that the affinities of the HAs of the A/Lyon-CHU/26430/03 and A/Reunion/586/04 H3NA- viruses may be low, to counterbalance the absence of NA activity. To verify this, we first measured the dissociation constant K_d_ of the A/Lyon-CHU/26430/03 and A/Reunion/586/04 natural isolates using biotinylated sialylglycopolymers (3′SL and 6′SLN) (Figure 1).

To analyse the results, the blank-corrected data were fitted with two non-linear regression models. The first model (MM model) corresponds to a classic Michaelis-Menten model and assumes that there is one site per receptor (i.e. monomer). The second model (MM2 model) is an adaptation of the Michaelis-Menten model with the assumption that there are two sites of different affinities per receptor. This model relates to the presence of a secondary sialic acid site in the HA, detected in crystallographic studies [19]. We use the Bayesian Information Criterion (BIC) to objectively discriminate between these two models.

We observed that the MM2 model was better for interpreting the results from binding assays with 6′SLN and 3′SL receptors for the A/Moscow/10/99 virus, implying that we may detect a signal resulting from the binding of sialylated receptors to the secondary site. For the A/Moscow/10/99 virus, the K_d_ constant given by the MM model and the K_d1_ constant by the MM2 model, representing the affinity of the primary sialic acid site in HA, i.e. receptor binding site (RBS), for sialylated receptors, are in the same range for both 6′SLN and 3′SL receptors, showing model coherence. The Kd_2_ constants detected in the MM2, representing affinity of the secondary sialic acid site in HA for sialylated receptors, is 20 and 13 times lower than the K_d1_ constants for 6′SLN and 3′SL, respectively. A better fit with the MM regression was obtained for the results of binding assays of the H3NA- viruses with 6′SLN receptors. This suggests that in those conditions, the secondary sialic acid site was not detected, maybe in relation to the low affinity of the primary sialic acid site. For 3′SL receptors, no MM2 regression could be obtain as a result of the low signal.

*In fine*, the A/Lyon-CHU/26430/03 and A/Reunion/586/04 H3NA- viruses possessed affinities for 6′SLN receptors respectively 49 and 128 times lower than the A/Moscow/10/99 virus. The affinities of the A/Lyon-CHU/26430/03 and A/Reunion/586/04 viruses for 3′SL receptors were hardly detectable and could not be interpreted even with the MM model, as binding could not be distinguished from the non-specific signal. The affinity of the A/Moscow/10/99 HA for 3′SL receptors is 20 times lower than for 6′SLN receptors.

### Generation of Recombinant Viruses Containing HA with Low Affinity

To test our hypothesis that a virus containing a deficient NA would be rescued when combined with an HA of decreased affinity for sialic acid receptors, we produced, by reverse genetics, recombinant viruses containing the deficient E119D/I222L NA and the HA of the A/Lyon-CHU/26430/03 (L/E119D/I222L) or A/Reunion/586/04 (R/E119D/I222L) H3NA- strains on the A/Moscow/10/99 genetic background. In parallel, construction of viruses containing the HAs of A/Lyon-CHU/26430/03 (L/H3NA-) and A/Reunion/586/04 (R/H3NA-) H3NA- strains without any NA segment on the A/Moscow/10/99 genetic background was attempted (Table 1). Two passages on MDCK cells were then performed.

**Table 1:** Recombinant viruses produced by reverse genetics and their growth characteristics after two passages in MDCK cells.

Virus name	Origin of the genes			Growth characteristics after the 2^nd^ passage in MDCK	
	Core^a^	HA	NA	CPE^b^	Log_10_ TCID_50_/50 µl^c^
L/E119D/I222L	A/Moscow/10/99	A/Lyon-CHU/26430/03	E119D/I222L A/Moscow/10/99	+++	5.4 +/–0,1
R/E119D/I222L	A/Moscow/10/99	A/Reunion/586/04	E119D/I222L A/Moscow/10/99	+++	4.7 +/–0
L/H3NA-	A/Moscow/10/99	A/Lyon-CHU/26430/03	No NA	-	<1
R/H3NA-	A/Moscow/10/99	A/Reunion/586/04	No NA	-	<1

a Core defined by the PB1, PB2, PA, NP, NS and M genes.

b CPE for cytopathic effect. – no visible CPE. +++ visible CPE.

c TCID_50_/50 µl : dose infecting 50% the cell culture determined in MDCK cells.

L/H3NA- and R/H3NA- viruses were not able to grow on MDCK cells as no cytopathic effect (CPE) and no infectivity (TCID_50_/50 µl) was detectable (Table 1). This implies that the low affinity HA alone is not sufficient to render the A/Moscow/10/99 virus independent of the NA gene. The two viruses R/E119D/I222L and L/E119D/I222L induced a CPE on MDCK cells. Their TCID_50_/50 µl were respectively 10^4.7^ and 10^5.4^.

### RNA Amplification

To verify that the E119D/I222L NA segments were incorporated in the virions, we performed plaques assays after the second passage on MDCK cells. Since the plaque morphologies were homogenous (1 mm), we extracted RNA from the viral stocks and specifically amplified the NA gene. After three passages in MDCK cells, the E119D/I222L mutation in the NA gene segment was shown to be preserved and no changes in sequence were detected. The HA genes of the R/E119D/I222L and L/E119D/I222L viruses were also analysed and no modifications were observed.

### Phenotypic Characteristics of NA

The P2 viruses were used to infect MDCK cells at a multiplicity of infection (m.o.i) of 0.001 and viruses were harvested 96 h post-infection. The viruses were concentrated through a saccharose cushion and then purified on a saccharose gradient. The purified viruses were standardized relative to the quantity of nucleoprotein (NP). The NA activities of the R/E119D/I222L and L/E119D/I222L viruses were measured by a fluorimetric assay using a synthetic MUNANA substrate. Under these conditions, no NA activity was detected either for the L/E119D/I222L or R/E119D/I222L virus whereas the NA activity of the A/Moscow/10/99 virus was of 603 nmol of MUNANA/h/ml (results from four independent assays). These results indicate that the combination of the E119D/I222L mutations abolishes the NA activity of the recombinant viruses. We also observed that the mutant NA was not detectable, using two monoclonal antibodies, in the L/E119D/I222L and R/E119D/I222L virions, or in 293T cells transfected with a plasmid containing the mutant NA (see Material and Methods S1, Results S1, Table S1 and Figures S1, S2 and S3).

### Kinetics in MDCK Cells

**Figure 2:**
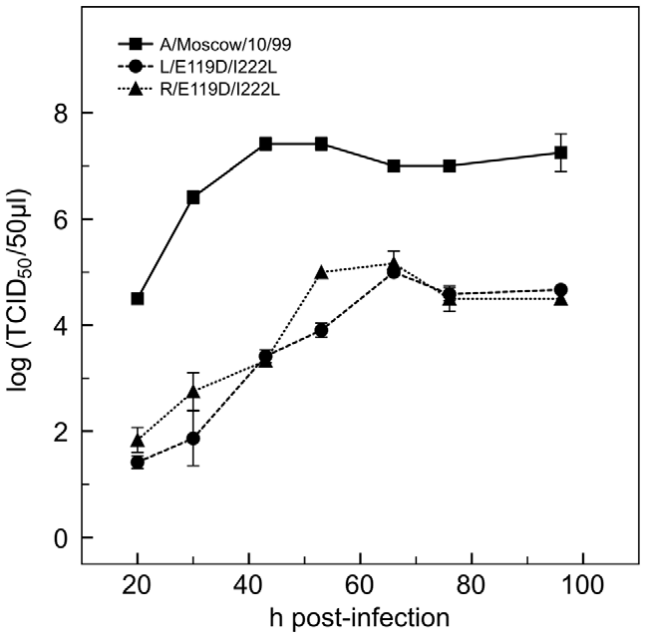
Kinetics of virus growth experiments in MDCK cells. P2 MDCK viruses were used to infect MDCK cells at a m.o.i of 0.001. Supernatants were harvested at the indicated times. The TCID_50_/50 µl values correspond to the mean +/– standard deviation calculated from two independent assays.

To evaluate the replicative capacities of the L/E119D/I222L and R/E119D/I222L viruses in comparison to the A/Moscow/10/99 virus, we monitored growth kinetics in MDCK cells by determining infectivity (TCID_50_/50 µl) over a 96 h time period (Figure 2).

Growth of the two R/E119D/I222L and L/E119D/I222L viruses was similar but the kinetics were slower than for the A/Moscow/10/99 virus. A plateau was reached at 60 h post-infection for both recombinant viruses at an infectivity titre about 2.5-log lower than for the A/Moscow/10/99 virus. At 96 h post-infection, some cellular monolayer remained implying that the recombinant viruses were not able to infect all the cells.

## Discussion

During 2003–2004, Ferraris et al. isolated four viruses lacking both a NA gene and protein [14]. Of these, two isolates A/Lyon-CHU/26430/03 and A/Reunion/586/04 (H3NA- viruses) were further analyzed and the HA gene was shown to be one of the principal determinants for the virus to be independent of the NA [15]. In this study, we showed that the HAs of these H3NA- viruses have reduced affinity for their receptors and hypothesized that this low affinity might compensate for deficiency in NA, activity and protein, of recombinant A/Moscow/10/99 (background) viruses.

The HAs of the two H3NA- viruses, A/Lyon-CHU/26430/03 and A/Reunion/586/04, possessed similar low affinities for 6′SLN receptors and no detectable affinity for 3′SL receptors. However, no mutations were observed in conserved residues of the RBS or elsewhere in the sequences of the HAs of the two H3NA- viruses, relative to contemporary viruses, which might account for altered binding. An alternative mechanism for the low HA affinity of the two H3NA- viruses could be the absence of desialylation of carbohydrate residues in the vicinity of the RBS, due to the total absence of NA activity, leading to a reduced accessibility for the receptor [20], [21]. Whatever the basis, the low HA affinity of these two natural isolates explains how these viruses are able to replicate in the absence of any detectable NA activity and protein.

The low receptor binding affinity of these HAs also allowed the rescue of an A/Moscow/10/99 recombinant virus containing a defective NA. The deficiency of the NA is due to a double mutation in framework sites (E119D/I222L), abolishing the replicative capacity of the recombinant A/Moscow/10/99 virus in MDCK cells in the absence of complementation by bacterial NA activity [14]. The L/E119D/I222L and R/E119D/I222L viruses had no detectable NA activity. The presence of the mutated E119D/I222L NA, both in transfected cells and in virion, was assessed using two different monoclonal antibodies and different techniques. Even though the results are not totally conclusive in the absence of a positive control for the detection of the mutant protein by the antibodies, our observations indicate that the mutation E119D/I222L might be deleterious for the expression of the NA, and consequently for NA activity. Moreover, using the HAs of the H3NA- viruses, we have been able to rescue, viruses containing a defective interfering NA RNA instead of the full NA gene (data not shown). Taken together, we have shown that the low affinity of the H3NA- virus HAs can compensate for the absence of both NA activity and of the NA protein. However, the L/H3NA- and R/H3NA- control viruses, containing the HA of the H3NA- viruses and six A/Moscow/10/99 virus genes, but lacking the NA gene, could not be rescued, in contrast to a complement of seven genes of the H3NA- viruses (Table 2). This implies that the low affinity of the HA alone is not sufficient to rescue the A/Moscow/10/99 genetic background in the absence of the NA gene and that other internal genes or proteins might play a role in the independence of the H3NA- viruses from the NA gene. Specific characteristics of the PB1 gene and/or protein might be implicated as the PB1 gene was found to be a determining factor, in addition to HA, for the “7 segment phenotype” [15].

**Table 2:** Experiments done on H3NA- viruses.

	Plasmid tranfected			Virus		Rescue	Study
Virus name	Core	HA	NA	NA			
				gene	protein		
E119D/I222L	A/Moscow/10/99	A/Moscow/10/99	E119D/I222L	N.D.	N.D.	no	[16]
L/E119D/I222L	A/Moscow/10/99	A/Lyon-CHU/26430/03	E119D/I222L	E119D/I222L	no	yes	this study
R/E119D/I222L	A/Moscow/10/99	A/Reunion/586/04	E119D/I222L	E119D/I222L	no	yes	this study
L/H3NA-	A/Moscow/10/99	A/Lyon-CHU/26430/03	No NA	no	no	no	this study
R/H3NA-	A/Moscow/10/99	A/Reunion/586/04	No NA	no	no	no	this study
H3NA-	A/Reunion/586/04	A/Reunion/586/04	No NA	no	no	yes	[15]

The NA plays a role in desialylating cellular and most importantly viral glycoproteins, enabling the progeny virions to avoid self-aggregation and to be released from host cells. The low HA affinity of the two H3NA- viruses allows the virus to be independent from NA activity. This implies that, even if the HA is not desialylated, the HA on its own dissociates from its receptor and the progeny virions do not aggregate.

Furthermore, the two natural H3NA- isolates A/Lyon-CHU/26430/03 and A/Reunion/586/04 did not exhibit reduction in replicative capacities in MDCK cells compared to A/Moscow/10/99 [14], in contrast to the L/E119D/I222L and R/E119D/I222L viruses. Whether the low replication of the latter viruses is due to reduced association of HA with sialylated receptors or a consequence of defective expression of the mutant NA, rather than its absence, is not evident from the available data. These results are however consistent with a role for internal genes/proteins, such as PB1, in complementing low HA affinity and absence of NA activity. Together, these observations emphasize the complexity in the interrelationships among different factors in optimizing the complementary activities of the HA and NA in virus replication.

## Materials and Methods

### Cells

Madin-Darby Canine Kidney (MDCK) cells used for production of viruses were purchased from Cambrex Bioscience (ATCC, CCL34) and were maintained in serum-free medium for MDCK cells (UltraMDCK, Lonza) supplemented with 1% L-Glutamine (L-Gln, 200 mM, Lonza) and 2% Penicillin-Streptomycin (PS, 10000 U Penicillin/ml, 10000 U Streptomycin/ml, Lonza).

MDCK cells used for reverse genetics were maintained in Minimum Essential Medium, Eagle’s with Earle’s Balanced Salt Solution without L-glutamine (EMEM, Lonza) supplemented with 1% L-Gln, 2% PS, 10% Foetal Bovine Serum (FBS, Lonza) and 1% Non Essential Amino Acids (NEAA 100X, Lonza).

293T cells, purchased from the ATCC (CRL-11-268), were maintained in Dulbecco’s modified Eagle’s medium with 4.5 g/l glucose without L-glutamine (DMEM) supplemented with 1% L-Gln, 2% PS and 10% FBS.

### Viruses

The isolates A/Lyon-CHU/26430/03, A/Reunion/586/04 and the A/Moscow/10/99 viruses were obtained from the National Reference Center (Lyon).

The A/Moscow/10/99 virus used in this study was produced by reverse genetics [16]. After verification of the homogeneity of the population, the fifth passage in MDCK cells was purified and used to perform the experiments.

We used an eight-plasmid system to generate recombinant viruses on the A/Moscow/10/99 background with combinations of the hemagglutinin genes from A/Lyon-CHU/26430/03 or A/Reunion/586/04 and the double mutant E119D/I222L neuraminidase gene from the A/Moscow/10/99 strain, as previously described [16]. Briefly, 8 segments cloned in the pHW2000 plasmid were used to transfect 293T cells in co-culture with MDCK cells, using Superfect Reagent. 72 h post-transfection, the supernatants were harvested and preserved at −80°C.

### Affinity of the Hemagglutinin for Sialic Acid Receptors

32 hemagglutination (HA) units of each virus in 50 µl of PBS were coated on 96-well plates (NUNC MAXISORP) over-night at room temperature in a humid atmosphere. Three five-minute washings with PBS, 0.05% Tween (Sigma) (130 µl/well) were then performed with gentle agitation at room temperature. Saturation by adding 100µl/well of Blocking Reagent (Roche) was for one hour at 37°C followed by three washings. In order to inhibit the NA activity, 96-well plates were incubated 30 min at 4°C and the remainder of the assay was done on ice using cold buffers. 50 µl/well of the biotinylated sialylglycopolymers Neu5Acα2-3Galß1-4Glc-PAA-Biotin (3′SL) and Neu5Acα2-6Galß1-4GlcNAc-PAA-Biotin (6′SLN) serially diluted two-fold in PBS, 0.05% Tween, 0,1% bovine serum albumin (BSA) and 10 µM zanamivir were added for 2 h at 4°C. Three five-minute washings with PBS 0.05% Tween, 10 µM of zanamivir (130 µl/well) were then performed with gentle agitation at room temperature. Anti-biotin antibody coupled to Horseradish Peroxidase (Euromedex) diluted in PBS, 0.05% Tween, 0,1% BSA, 10 µM zanamivir (50 µl/well) was then added and incubated 2 h at 4°C followed by three five-minute washings with PBS, 0.05% Tween, 10 µM of zanamivir (130 µl/well) with gentle agitation at room temperature. The peroxidase reaction with TMB substrate (0.2 g/l of Tetramethylbenzidine, KPL) for 5 min with shaking in the dark was stopped with 50 µl/well of phosphoric acid at 0,8 mol/l. Optical density at 450 nm was determined with a microplate reader (UMV 340 Asys, BioServ).

Blank-corrected data were plotted and fitted with two established binding models by non-linear least-squares regressions with R software. The first model was the Michaelis-Menten equation for reversible monovalent binding (MM model [22]), based on one site per receptor and negligible ligand depletion. The equation was the following:
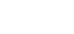
with r_0_, the number of sites and K_d_, the dissociation constant, both parameters to be fitted.

The second model was an adaptation of the Michaelis-Menten model (MM2 model) based on two sites of different affinities per receptor. The resulting equation was the sum of two different MM models, one for each site subpopulation:
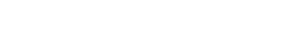



Assuming that receptors are bivalent, the total number of sites of both types is the same, leaving three parameters to be fitted : r_0_, Kd_1_ and Kd_2_.

We used the Bayesian Information Criterion (BIC), a tool able to discriminate among a class of parametric models with different numbers of parameters [23]. The BIC is very sensitive to overfitting as it introduces a penalty term for the number of parameters in the model.
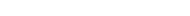
with n, the number of observations, k, the number of parameters and s the error variance. When comparing different models for the same data, the smaller the criterion value, the better the model. If our test allows us to detect the secondary site, the MM2 model should turn out to be the better model.

### Passage on MDCK Cells

Viruses produced by reverse genetics were passaged two times in MDCK cells. For the first passage, confluent MDCK cells were inoculated with 10-fold dilutions of viruses in EMEM, 2% PS, 1% L-Gln, 1 µg/ml of trypsin (Roche) (working medium) for one hour at 34°C. Fresh working medium was then added to the cultures and supernatants were harvested after varying times, depending on the appearance of cytopathic effect to ensure a good amplification of virus. For the second passage, 0.01 m.o.i were used to infect confluent MDCK monolayers. The inoculum was withdrawn and fresh working medium added to the culture. The viruses were harvested 72 h post-infection to allow comparison of the replicative capacities.

### Purification of Viruses

Second passage (P2) viruses were used to infect MDCK cells at a m.o.i of 0.001. Supernatants were harvested depending on the cytopathic effect and centrifuged at 1800 *g* for 10 min to eliminate cellular debris. Viruses were then concentrated through a 25% (w/w) saccharose cushion by ultracentrifugation for 2 h at 126000 *g*. Pellets were resuspended in HBS-N buffer (0.01M HEPES, 0.15M NaCl, pH = 7.4) and centrifuged through a linear gradient of saccharose (22%–60% (w/w)) for 14 h at 260000 *g* without brake. Bands of purified viruses were harvested and saccharose was removed by diluting the viruses ten times in PBS and by centrifugating at 126000 *g* for 2 h. The final pellets were resuspended in HBS-N buffer, aliquoted and preserved at −80°C. The total amount of viral protein was determined using a Micro BCA Protein Assay Kit (Thermo Scientific, Pierce), according to the manufacturer’s instructions.

### Infectivity Titration

Infectious titers (TCID_50_/50 µl), were determined on confluent MDCK cells as previously described [16]. Briefly, tenfold serial dilutions of viruses were inoculated in quadruplicate onto MDCK cells. Endpoints were determined by HA tests performed with 0.8% guinea pig red blood cells for one hour at room temperature. TCID_50_/50 µl were calculated by the Reed and Muench test [24].

### Plaque Assays

Confluent MDCK cell monolayers were incubated for one hour with tenfold serial dilutions of P2 MDCK viruses in working medium. The supernatants were withdrawn and the cells were overlaid with freshly prepared EMEM (2x concentrate, Lonza) containing 0.55% agar (Agar Noble, Difco) and 1 µg/ml of trypsin. The plaques were visualized 72 h post-infection after staining with a solution containing 0,7% agar and 0.02% neutral red (Sigma) in PBS.

### HA and NA Sequencing

RNA extracted from the viral stocks was reverse transcribed and the NA and HA genes were specifically amplified and sequenced (GATC Biotech). Sequences were analysed by SeqMan software.

### NA Activity Assays

Fluorimetric NA activity assays were done as described by Ferraris et al. [14]. Total NA activities were calculated as the quantity of MUNANA substrate degraded in one hour per ml of virus suspensions (nmol/h/ml).

### Kinetics of Growth in MDCK Cells

Confluent MDCK cells monolayers were infected for one hour with the appropriate dilutions of viruses, at a m.o.i of 0.001 in a minimum volume of working medium. The supernatants were withdrawn and the cells were washed two times with PBS and then overlaid with working medium. At the indicated times, supernatants were harvested and frozen at -80°C. Infectious titers of each sample were determined on confluent MDCK cells as described in the preceding section.

## Supporting Information

Material and Methods S1 **Quantification of the expression of wild type and E119D/I222L NAs in transfected 293T by flow cytometry.** Quantification of wild type and E119D/I222L NAs in virions using an ELISA-based assay.
(DOCX)

Results S1 **Expression of wild type and E119D/I222L NAs in transfected 293T and at their cell membrane.** Quantity of NA in L/E119D/I222L and R/E119D/I222L virions.
(DOCX)

Figure S1 **Flow cytometry of transfected non-permeabilized stained 293T cells.** The gate P3 represents the number of fluorescent cells. A. Staining with the M9G3D5 mouse monoclonal antibody and FITC-A labelled goat anti-mouse IgG antibody. 293T cells transfected with A1, NA A/Moscow/10/99 wild type, A2, NA A/Moscow/10/99 E119D/I222L and A3, pHW2000. B. Staining with the M6G5D6 mouse monoclonal antibody and FITC-A labelled goat anti-mouse IgG antibody. 293T cells transfected with B1, NA A/Moscow/10/99 wild type, B2, NA A/Moscow/10/99 E119D/I222L and B3, pHW2000. C. Staining with the NR-4540 mouse monoclonal antibody and FITC-A labelled goat anti-mouse IgG antibody. 293T cells transfected with C1, PR8 and C2, pHW2000.
(TIF)

Figure S2 **Flow cytometry of transfected permeabilized stained 293T cells.** The gate P3 represents the number of fluorescent cells. A. Staining with the M9G3D5 mouse monoclonal antibody and FITC-A labelled goat anti-mouse IgG antibody. 293T cells transfected with A1, NA A/Moscow/10/99 wild type, A2, NA A/Moscow/10/99 E119D/I222L and A3, pHW2000. B. Staining with the M6G5D6 mouse monoclonal antibody and FITC-A labelled goat anti-mouse IgG antibody. 293T cells transfected with B1, NA A/Moscow/10/99 wild type, B2, NA A/Moscow/10/99 E119D/I222L and B3, pHW2000. C. Staining with the NR-4540 mouse monoclonal antibody and FITC-A labelled goat anti-mouse IgG antibody. 293T cells transfected with C1, PR8 and C2, pHW2000. D. 293T cells transfected with D2, EGFP-N1 and D3, pHW2000
(TIF)

Figure S3 **NA protein detection in A/Moscow/10/99, L/E119D/I222L and R/E119D/I222L viruses.** The NA protein was detected by an ELISA-based assay using a monoclonal antibody directed against the NA of A/Moscow/10/99. Blank-corrected data result from three independent assays and were plotted against A/Moscow/10/99 virus total protein.
(TIFF)

Table S1 **Expression of different NAs in 293T cells determined by flow cytometry after permeabilization, or not, of the cell membrane.**
(DOCX)
