# Supplementary material for: Rescue of a H3N2 Influenza Virus Containing a Deficient Neuraminidase Protein by a Hemagglutinin with a Low Receptor-Binding Affinity
Source: PLoS One. 2012 May 1;7(5):e33880. doi: 10.1371/journal.pone.0033880 (PMC3341378; doi:10.1371/journal.pone.0033880)
Supplement: Material and Methods S1 — Quantification of the expression of wild type and E119D/I222L NAs in transfected 293T by flow cytometry. Quantification of wild type and E119D/I222L NAs in virions using an ELISA-based assay. (DOCX) [file pone.0033880.s001.docx]

**Material and Methods S1**

**Flow cytometry.**

Transient calcium phosphate-mediated transfections of 293T cells were performed essentiality as previously described [1]. 293T cells were co-transfected with 1µg of each polymerase complex genes of A/Puerto-Rico/8/34 (PR8) cloned in pCAGGS and 1µg of respectively the following plasmids : NA A/Moscow/10/99 wild type, NA A/Moscow/10/99 E119D/I222L and NA PR8 cloned in pHW2000, empty pHW2000 and the eGFP-N1 flanked by the NCRs from the segment 8 of PR8 [1]. To determine the percentage of transfection, cells were transfected with 4µg of eGFPN1 (Clonetech, BD Bioscience, Amsterdam, The Netherlands) or 4µg of pCDNA3. Fluorescent cells were counted by a FACSCantoII and the amount of fluorescent cells, corrected for background signal, gave a percentage of transfection of 93.5%.

24 h post-transfection, cells were harvested and stained with **LIVE⁄DEAD® Fixable Dead Cell Stain Kit** (Invitrogen) according to the manufacturer’s intructions. Cells were then washed and fixed with BD Cytofix™ overnight at 4°C. To permeabilize the cell membrane, cells were washed twice with BD Cytoperm™, diluted 1/10 in H_2_O. Cells were then stained for 45 min at 4°C with the following antibodies : M9G3D5 and M6G5D6, mouse monoclonal IgG antibodies raised against the NA of A/Moscow/10/99 (produced in the VirPath laboratory) and the NR-4540 (Beiressources) mouse monoclonal IgG antibody raised against the NA of PR8. After a washing step, cells were stained with a fluorescein isothiocyanate (FITC-A) labelled goat anti-mouse IgG antibody (BD) for 45 min at 4°C. Cells were then washed twice and analysed by flow-cytometry with a FACSCantoII and FACS Diva software (BD).

**Quantification of NA proteins.**

Total NA protein was quantified using an enzyme-linked immunosorbent assay (ELISA) as described previously using the mouse IgG monoclonal antibody M9G3D5 [2]. Purified viruses were standardized against the quantity of NP contained in 0.5 µg of total A/Moscow/10/99 virus protein, using an anti-NP monoclonal antibody (produced in the VirPath laboratory). Optical density at 405 nm was determined using a microplate reader (UMV 340 Asys, BioServ) and blank-corrected data were plotted.

1. de Wit E, Spronken MI, Bestebroer TM, Rimmelzwaan GF, Osterhaus AD, et al. (2004) Efficient generation and growth of influenza virus A/PR/8/34 from eight cDNA fragments. Virus Res 103: 155-161.

2. Richard M, Ferraris O, Erny A, Barthelemy M, Traversier A, et al. (2011) Combinatorial effect of two E119V+I222L framework mutations in the neuraminidase active site of H3N2 influenza virus on resistance to oseltamivir. Antimicrob Agents Chemother.
