# Supplementary material for: Rescue of a H3N2 Influenza Virus Containing a Deficient Neuraminidase Protein by a Hemagglutinin with a Low Receptor-Binding Affinity
Source: PLoS One. 2012 May 1;7(5):e33880. doi: 10.1371/journal.pone.0033880 (PMC3341378; doi:10.1371/journal.pone.0033880)
Supplement: Results S1 — Expression of wild type and E119D/I222L NAs in transfected 293T and at their cell membrane. Quantity of NA in L/E119D/I222L and R/E119D/I222L virions. (DOCX) [file pone.0033880.s002.docx]

**Absence of detection of the E119D/I222L NA in transfected 293T cells and in R/E119D/I222L and L/E119D/I222L viruses using two monoclonal antibodies.**

In a previous study, we were able to rescue and grow viruses containing the E119D/I222L mutation in the NA active site only in the presence of exogenous bacterial NA. These results suggest that the mutation E119D/I222L has a deleterious effect on the NA activity. To investigate if this mutation also has an effect on the NA expression in the cell and at the cell membrane, we transfected both the wild type NA and the E119D/I222L NA in 293T cells along with the polymerase complex of PR8. 24 h post-tranfection, the cells were harvested, stained with two different monoclonal antibodies raised against the NA of A/Moscow/10/99 (M9G3D5 and M6G5D6) and analysed by flow cytometry. As a positive control, the NA of PR8 was also transfected and cells were stained with a specific monoclonal antibody. The activity of the polymerase complex was determined by its co-transfection with a reporter gene containing the eGPFN1 flanked by the NCRs of the non-structural protein (NS) gene from PR8.

The eGFPN1 was expressed in 23.1% the cells (Table S1 and Figures S1 and S2). The NA of PR8 was detected in 12.2% the cells and the NA from A/Moscow/10/99 in 2.7% and 4.7% the cells using respectively M9G3D5 and M6D5G6 monoclonal antibodies. Using both monoclonal antibodies, the E119D/I222L mutant was not detected, either in the cell or at the cell membrane.

The quantity of NA present in the R/E119D/I222L and L/E119D/I222L viruses was measured by an ELISA assay using the monoclonal antibody M9G3D5 (Figure S3). No NA protein was detectable in the R/E119D/I222L or L/E119D/I222L virions. This result is consistent with the absence of detection of the E119D/I222L mutant after the transfection of 293T cells.

Although not conclusive about the expression of the mutant NA because of the lack of a positive control for detection of the mutant NA, these observations suggest that the mutation E119D/I222L might not only have a deleterious impact on the NA activity but also on the expression of the NA protein.
