# Supplementary figures and images for: Rescue of a H3N2 Influenza Virus Containing a Deficient Neuraminidase Protein by a Hemagglutinin with a Low Receptor-Binding Affinity
Source: PLoS One. 2012 May 1;7(5):e33880. doi: 10.1371/journal.pone.0033880 (PMC3341378; doi:10.1371/journal.pone.0033880)

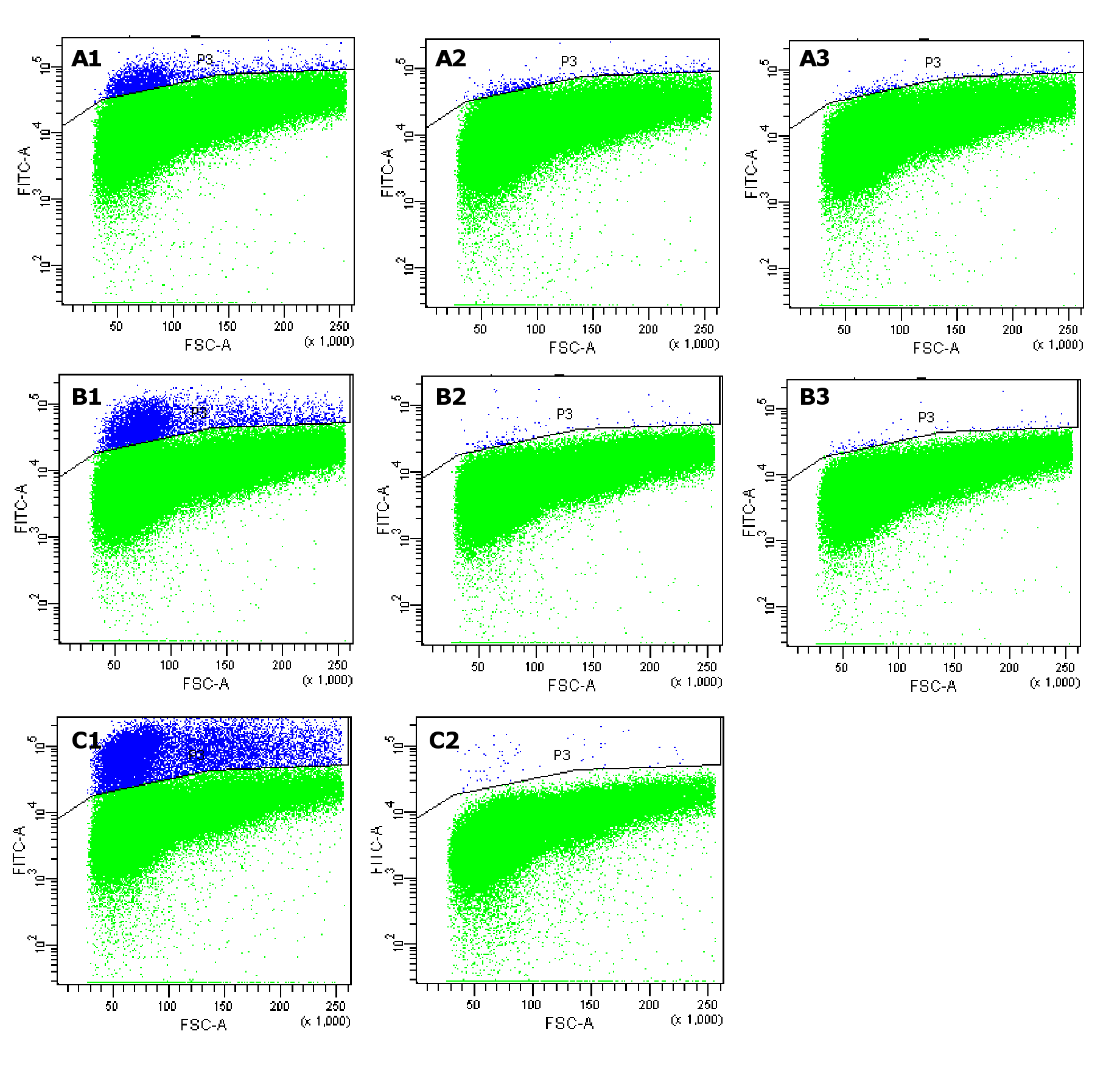

Supplement: Figure S1 — Flow cytometry of transfected non-permeabilized stained 293T cells. The gate P3 represents the number of fluorescent cells. A. Staining with the M9G3D5 mouse monoclonal antibody and FITC-A labelled goat anti-mouse IgG antibody. 293T cells transfected with A1, NA A/Moscow/10/99 wild type, A2, NA A/Moscow/10/99 E119D/I222L and A3, pHW2000. B. Staining with the M6G5D6 mouse monoclonal antibody and FITC-A labelled goat anti-mouse IgG antibody. 293T cells transfected with B1, NA A/Moscow/10/99 wild type, B2, NA A/Moscow/10/99 E119D/I222L and B3, pHW2000. C. Staining with the NR-4540 mouse monoclonal antibody and FITC-A labelled goat anti-mouse IgG antibody. 293T cells transfected with C1, PR8 and C2, pHW2000. (TIF) [file pone.0033880.s003.tif]

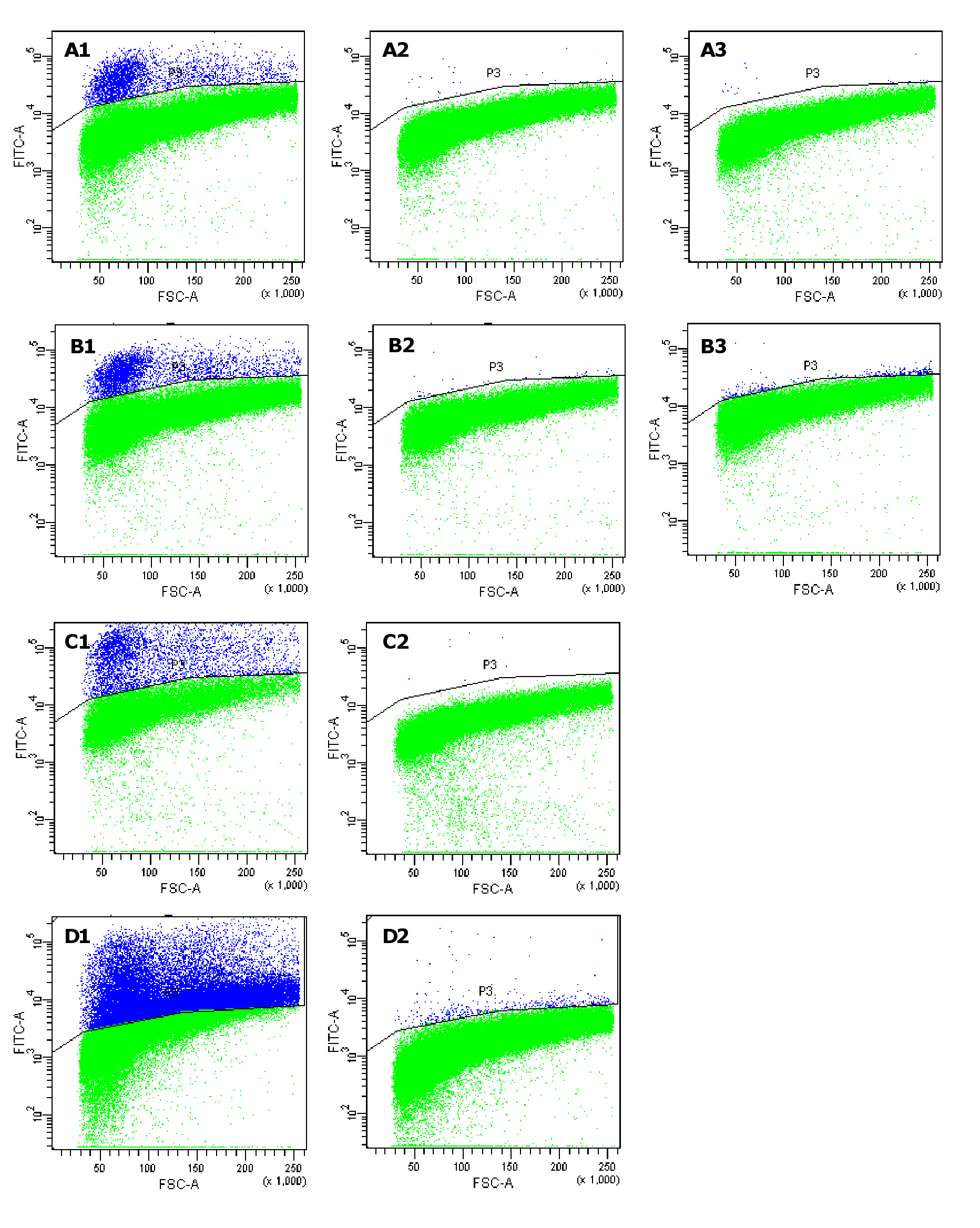

Supplement: Figure S2 — Flow cytometry of transfected permeabilized stained 293T cells. The gate P3 represents the number of fluorescent cells. A. Staining with the M9G3D5 mouse monoclonal antibody and FITC-A labelled goat anti-mouse IgG antibody. 293T cells transfected with A1, NA A/Moscow/10/99 wild type, A2, NA A/Moscow/10/99 E119D/I222L and A3, pHW2000. B. Staining with the M6G5D6 mouse monoclonal antibody and FITC-A labelled goat anti-mouse IgG antibody. 293T cells transfected with B1, NA A/Moscow/10/99 wild type, B2, NA A/Moscow/10/99 E119D/I222L and B3, pHW2000. C. Staining with the NR-4540 mouse monoclonal antibody and FITC-A labelled goat anti-mouse IgG antibody. 293T cells transfected with C1, PR8 and C2, pHW2000. D. 293T cells transfected with D2, EGFP-N1 and D3, pHW2000 (TIF) [file pone.0033880.s004.tif]

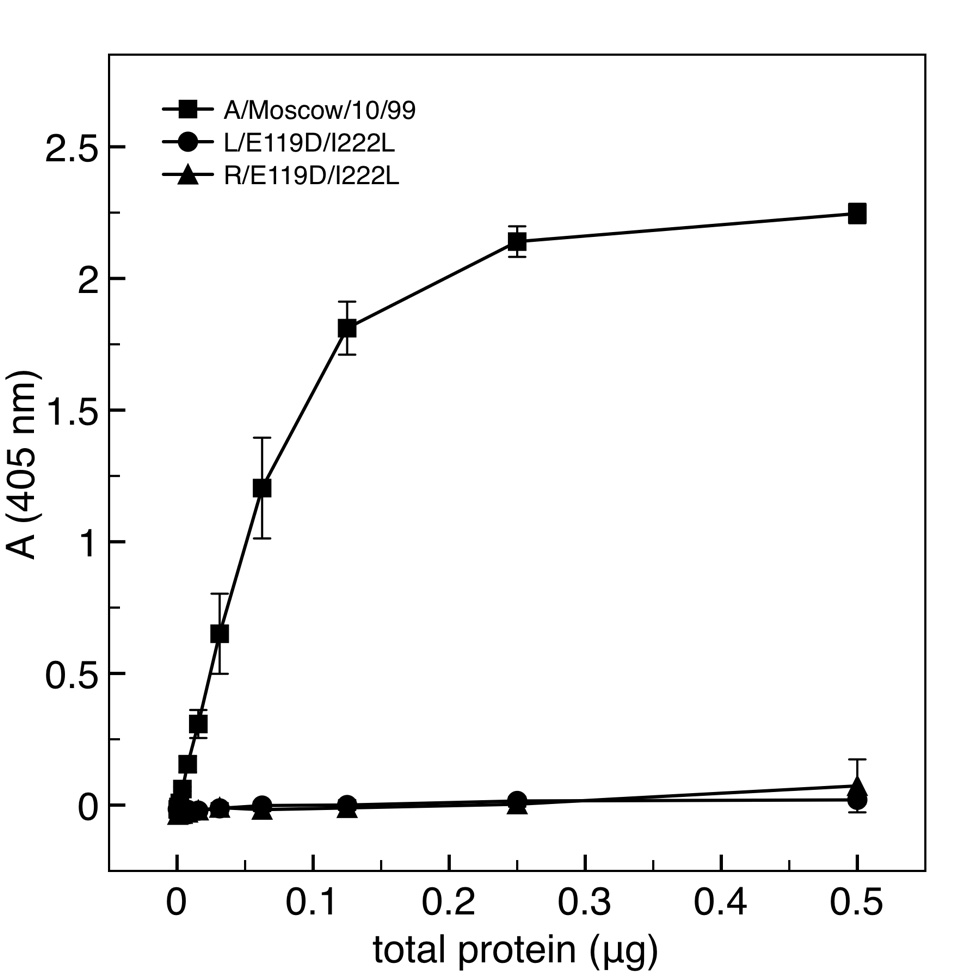

Supplement: Figure S3 — NA protein detection in A/Moscow/10/99, L/E119D/I222L and R/E119D/I222L viruses. The NA protein was detected by an ELISA-based assay using a monoclonal antibody directed against the NA of A/Moscow/10/99. Blank-corrected data result from three independent assays and were plotted against A/Moscow/10/99 virus total protein. (TIFF) [file pone.0033880.s005.tif]
