# Supplementary material for: Rescue of a H3N2 Influenza Virus Containing a Deficient Neuraminidase Protein by a Hemagglutinin with a Low Receptor-Binding Affinity
Source: PLoS One. 2012 May 1;7(5):e33880. doi: 10.1371/journal.pone.0033880 (PMC3341378; doi:10.1371/journal.pone.0033880)
Supplement: Table S1 — Expression of different NAs in 293T cells determined by flow cytometry after permeabilization, or not, of the cell membrane. (DOCX) [file pone.0033880.s006.docx]

| Plasmid^a^ | Antibody | Treatment of the cells | |
| --- | --- | --- | --- |
|  |  | Permeablized | Not permeabilized |
|  |  | Percentage of fluorescent cells | |
| NA A/Moscow/10/99 | M9G3D5 | 2 | 2,7 |
| NA E119D/I222L | M9G3D5 | 0 | 0,3 |
| pHW2000 | M9G3D5 | 0 | 0,2 |
| NA A/Moscow/10/99 | M6G5D6 | 4,7 | 3,9 |
| NA E119D/I222L | M6G5D6 | 0,1 | 0,1 |
| pHW2000 | M6G5D6 | 0,6 | 0,1 |
| NA PR8 | NR-4540 | 12,2 | 10,3 |
| pHW2000 | NR-4540 | 0 | 0,1 |
| pEGFP-N1 | - ^b^ | 23,1 | - ^b^ |
| pHW2000 | - ^b^ | 0,1 | - ^b^ |

^a^ All the plasmids presented in this table were co-transfected in 293T cells with pCAGGS plasmids containing the genes of the polymerase complex of PR8 (PB2, PB1, PA, NP).

^b^ - : Not applicable
